# UV hyper-resistance in *Prochlorococcus* MED4 results from a single base pair deletion just upstream of an operon encoding nudix hydrolase and photolyase

**DOI:** 10.1111/j.1462-2920.2010.02203.x

**Published:** 2010-07

**Authors:** Marcia S Osburne, Brianne M Holmbeck, Jorge Frias-Lopez, Robert Steen, Katherine Huang, Libusha Kelly, Allison Coe, Kristin Waraska, Andrew Gagne, Sallie W Chisholm

**Affiliations:** 1 Department of Civil and Environmental Engineering, Massachusetts Institute of TechnologyCambridge, MA 02139, USA; 2 Biopolymers Facility, Harvard Medical SchoolBoston, MA 02115, USA

## Abstract

Exposure to solar radiation can cause mortality in natural communities of pico-phytoplankton, both at the surface and to a depth of at least 30 m. DNA damage is a significant cause of death, mainly due to cyclobutane pyrimidine dimer formation, which can be lethal if not repaired. While developing a UV mutagenesis protocol for the marine cyanobacterium *Prochlorococcus*, we isolated a UV-hyper-resistant variant of high light-adapted strain MED4. The hyper-resistant strain was constitutively upregulated for expression of the *mutT*-*phrB* operon, encoding nudix hydrolase and photolyase, both of which are involved in repair of DNA damage that can be caused by UV light. Photolyase (PhrB) breaks pyrimidine dimers typically caused by UV exposure, using energy from visible light in the process known as photoreactivation. Nudix hydrolase (MutT) hydrolyses 8-oxo-dGTP, an aberrant form of GTP that results from oxidizing conditions, including UV radiation, thus impeding mispairing and mutagenesis by preventing incorporation of the aberrant form into DNA. These processes are error-free, in contrast to error-prone SOS dark repair systems that are widespread in bacteria. The UV-hyper-resistant strain contained only a single mutation: a 1 bp deletion in the intergenic region directly upstream of the *mutT*-*phrB* operon. Two subsequent enrichments for MED4 UV-hyper-resistant strains from MED4 wild-type cultures gave rise to strains containing this same 1 bp deletion, affirming its connection to the hyper-resistant phenotype. These results have implications for *Prochlorococcus* DNA repair mechanisms, genome stability and possibly lysogeny.

## Introduction

*Prochlorococcus*, a marine cyanobacterium that numerically dominates the mid-latitude oceans, is the smallest known oxygenic phototroph and plays a central role in the oceanic carbon cycle. Its numbers can reach as high as 10^5^ cells ml^−1^. The group consists of genetically distinct ecotypes that can be broadly classified as high or low light-adapted, depending on their optimal light intensity for growth (reviewed in Coleman and Chisholm, 2007). The high light-adapted ecotypes are found throughout the euphotic zone of the oceans (down to a depth of 150–200 m), reaching highest abundance in surface waters, whereas their low light-adapted counterparts dominate deeper waters.

Factors that affect mortality of *Prochlorococcus* include grazing, mainly from phagotrophic flagellates and microzooplankton (Guillou *et al.*, 2001; Christaki *et al.*, 2002; Frias-Lopez *et al.*, 2008), and phage infection (Sullivan *et al.*, 2003). In addition, for cells close to the surface, exposure to high solar radiation, especially in the UVB range (280–320 nm), also plays a role in mortality both at the surface and to a depth of 10–30 m (Llabrés and Agustí, 2006; Agustí and Llabrés, 2007). DNA damage, mainly due to the formation of cyclobutane pyrimidine dimers, can be lethal if not repaired and is a primary cause of UV-induced mortality (Sancar, 2000). The high light-adapted *Prochlorococcus* ecotypes that dominate surface waters have very small (1.66–1.74 Mb), streamlined, non-redundant genomes (Strehl *et al.*, 1999; Kettler *et al.*, 2007), making them potentially less able to withstand the effects of multiple mutations resulting from high doses of UV light. It is of interest therefore to determine whether the high and low light-adapted ecotypes have different susceptibilities to UV damage, and the mechanisms that might mediate these susceptibilities.

In the process of developing a mutagenesis protocol for *Prochlorococcus* using UV light (M.S. Osburne and B.M. Holmbeck, in prep.) we isolated a UV-hyper-resistant strain (MED4 UVR1) of high light-adapted strain *Prochlorococcus* MED4. We examined the differences between the hyper-resistant and wild-type (WT) cells with respect to levels of UV resistance, gene expression and genome sequence. The results allowed us to propose a mechanism for UV hyper-resistance in MED4 UVR1, and to hypothesize mechanisms by which MED4 copes with UV light in its natural environment.

## Results

### Isolation and initial phenotypic characterization of MED4 UV-hyper-resistant strains

**Fig. 1:**
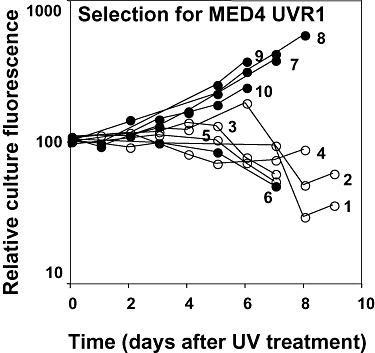
Enrichment selection for UV-hyper-resistant *Prochlorococcus* MED4 (MED4 UVR1). Cells from a mid-log phase culture of MED4 WT were subjected to 30 s of UV exposure (254 nm) on day 0, then returned to standard growth conditions. This was done for 10 consecutive rounds of UV exposure and regrowth (labelled 1–10). ○, rounds 1–5; •, rounds 6–10.

A culture of high light-adapted *Prochlorococcus* strain MED4 was subjected to 10 rounds of UV treatment in which cells were grown to mid-log phase, treated with UV light (*Experimental procedures*), and allowed to recover under standard growth conditions in visible light. Recovery of culture fluorescence after UV treatment occurred more readily with increasing rounds of treatment. After 7–8 rounds of treatment and transfer, the cultures were significantly more resistant to UV light than the parent strain, as reflected in the selection for the UV-hyper-resistant strain MED4 UVR1 (Fig. 1). This general pattern was also seen during similar enrichment selections (not shown) for two additional UV-hyper-resist strains (MED4 UVR2 and MED4 UVR3), carried out at a later date. To confirm that the decrease in fluorescence caused by UV treatment was due to cell lysis, we showed that culture fluorescence of WT and hyper-resistant cells remained proportional to cell number for both UV-treated and untreated cells (Fig. S1),

### UV resistance

**Fig. 2:**
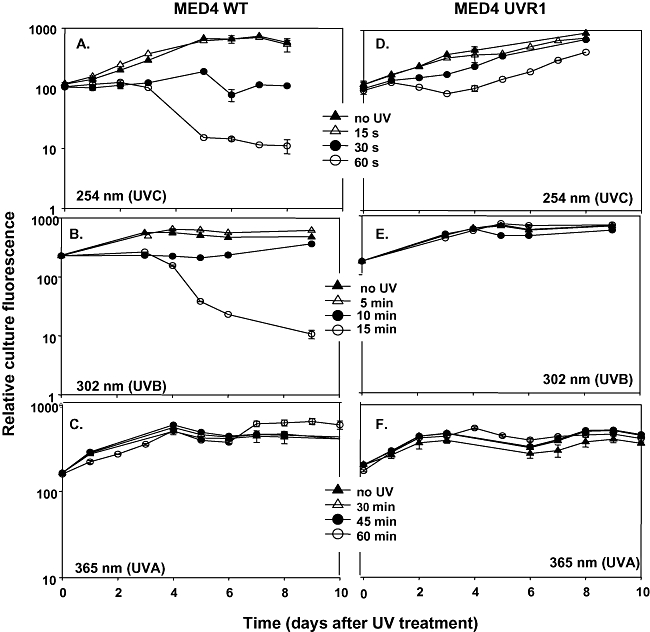
Sensitivity to UV light of MED4 WT and MED4 UVR1. MED4 WT (A, B, C) and MED4 UVR1 (D, E, F) were treated with UV light at three wavelengths: 254, 302 and 365 nm (UVC, UVB and UVA respectively), for various time intervals on day 0. Following UV treatment, cells were incubated under standard growth conditions and monitored over time, using relative culture fluorescence as a measure of cell density in the cultures. Values are the mean of measurements of duplicate cultures, with error bars showing the standard deviation.

Cultures of MED4 WT and MED4 UVR1 were grown and tested for relative sensitivity to UV light at three different wavelengths: 254, 302 and 365 nm (UVC, UVB and UVA respectively). At equivalent doses of UV light, MED4 UVR1 (Fig. 2D–F) was considerably more resistant to both UVC and UVB than MED4 WT (Fig. 2A–C). However, both the hyper-resistant and WT strains were equally insensitive to UVA at the doses used in this experiment. While UVC and UVB are known to be absorbed by and cause direct damage to DNA, UVA is poorly absorbed by DNA, and its effect on cells is thought to be more complex and indirect (Kidambi *et al.*, 1996).

### Growth rates of MED4 WT and MED4 UVR1

To assess whether acquisition of the UV-hyper-resistant phenotype affected the growth rate under standard conditions in the absence of UV light, we measured the growth rates of WT and resistant strains in exponential phase. For MED4 WT, µ was 0.64 ± 0.01 day^−1^, compared with 0.67 ± 0.01 day^−1^ for MED4 UVR1, a significant difference according to the two tailed *t*-test using independent samples (*P* < 0.05). When the experiment was repeated, the growth rates were not significantly different, leading us to conclude that any such difference between the two strains under these conditions is small, and can only be revealed by very precise and repeated growth rate measurements. We cannot rule out that the growth rates of these strains may well vary significantly under conditions prevalent in their natural environment, where UV light is frequently present and other selection pressures may apply.

### Gene expression

**Fig. 3:**
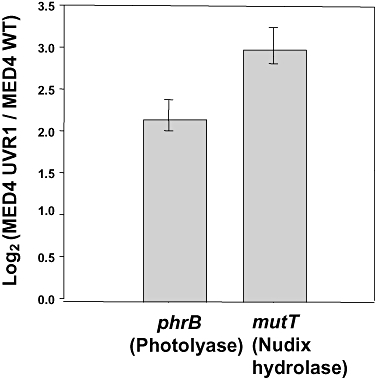
Expression of *phrB* and *mutT* in MED4 UVR1 relative to MED4 WT in log phase cells without UV treatment. Expression of *phrB* (encoding photolyase) and *mutT* (encoding nudix hydrolase) was measured using whole genome microarrays. These were the only genes that were significantly differentially expressed between MED4 WT and MED4 UVR1. Ratios are the mean values ± standard error for two microarray experiments, each carried out in duplicate.

To begin to explore the underlying mechanisms mediating UV hyper-resistance in MED4 UVR1 we compared the complete transcriptomes of MED4 WT and MED4 UVR1 cells in mid log-phase under standard growth conditions in the absence of UV treatment, using whole genome microarrays. The only significant difference seen was in the expression of two genes, *phrB* and *mutT*, encoding photolyase and nudix hydrolase respectively, which were more highly expressed in MED4 UVR1 relative to MED4 WT ( Fig. 3). Interestingly, these two genes form a small predicted operon in MED4 WT, consistent with their apparent co-regulation in MED4 UVR1. These genes, including their order and context between the *folK* and *degT* genes, are conserved in all the sequenced high light-adapted strains of *Prochlorococcus*, and also in strains NATL1A and NATL2A, which show increased fitness in variable light (http://www.microbesonline.org, Kettler *et al.*, 2007).

We next used qRT-PCR to analyse a time-course of expression of several selected genes following UV treatment (primers shown in Table 1), guided in our choice of genes by the expression results from untreated MED4 WT and MED4 UVR1 cells described above. In addition to *mutT* and *phrB*, already shown to be constitutively upregulated in MED4 UVR1 cells, *w*e examined the expression of DNA repair-related genes *recA* and *radA,* that might also be involved in the cellular response to UV light: *recA* is known to play a pivotal role in response to bacterial DNA damage (Michel, 2005), and while the *recA* analogue *radA* is of uncertain function in bacteria, it is thought to be involved in recombinational repair of DNA at replication forks blocked due to DNA damage [possibly resulting from pyrimidine dimer formation (Beam *et al.*, 2002)]. *radA* is also known to be upregulated in response to UVB and UVC radiation in the archaeon *Halobacterium* sp. NRC-1 (Boubriak *et al.*, 2008).

**Table 1:** Primers used for qRT-PCR reactions.

Gene	Primers
*phrB*	Forward: 5′-TGCAAGTTCAAGAGCTTGGTT-3′
	Reverse: 5′-CGGATCTCCTTCTTCCAAAA-3′
*mutT*	Forward: 5′-AACTCTTGTCCCTGCTCCTG-3′
	Reverse: 5′-TCTCCTTTAACCTCGGAATTGA-3′
*recA*	Forward: 5′-CTTGGAGATGCCTCCAGAAT-3′
	Reverse: 5′-CAACCACTCTTCCCTTTGGA-3′
*radA*	Forward: 5′-GAAGGATCGAGACCATTTGC-3′
	Reverse: 5′-GCTCATTCCAGTTGTTGTTCG-3′
*rnpB*	Forward: 5′GAGGATAGTGCCACAGAAACATACC-3′
	Reverse: 5′-GCTGGTGCGCTCTTACCACACCCTTG-3′

**Fig. 4:**
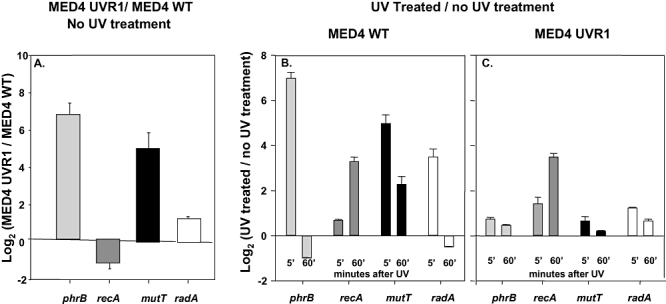
Expression of DNA repair genes determined by qRT-PCR in MED4 UVR1 and MED4 WT, with and without UV treatment. Ratios are the mean values ± standard error for two independent PCR runs, each carried out in duplicate. A. Transcript levels of four genes in MED4 UVR1 relative to MED4 WT in log phase cells in the absence of UV treatment. B and C. Transcript levels of the same four genes in log phase cultures subjected to brief UV treatment (254 nm, 30 s), relative to untreated controls. Transcript levels at 5 and 60 min after UV treatment are shown for MED4 WT (B) and MED4 UVR1 (C).

In the absence of UV treatment, in steady-state growth, MED4 UVR1 again had considerably higher levels of *phrB* and *mutT* transcripts relative to MED4 WT (Fig. 4A), in addition to slight constitutive upregulation of *radA* that was below the level of detection in the microarray experiment. Under the same conditions, *recA* expression was slightly lower in MED4 UVR1 as compared with MED4 WT, possibly a consequence of the constitutively high level of *phrB* and *mutT* expression (for example, excessive amounts of PhrB and MutT may reduce background levels of DNA damage, thus decreasing the need for RecA function).

To explore the dynamics of the expression of these genes in response to UV exposure, we compared transcript levels of UV-treated and untreated MED4 WT and MED4 UVR1 cells 5 and 60 min after treatment (Fig. 4B and C). For MED4 WT, *phrB*, *mutT* and *radA* were rapidly upregulated, showing elevated transcript levels by 5 min post treatment. By 60 min post treatment the levels dropped – in two cases below those of the untreated cells, suggesting the need for their function had diminished. In contrast, for MED4 UVR1, *phrB*, *mutT and radA* were only slightly upregulated following UV irradiation, with transcripts decreasing only slightly between 5 and 60 min, consistent with their constitutively elevated levels. The kinetics of upregulation of the *phrB*-*mutT* operon in the WT strain (which, although rapid, takes some time as compared with constitutive expression in the mutant) provides an explanation for increased UV resistance in the mutant strain, i.e. its constitutively upregulated enzymes are already present and poised to repair damage before the onset of UV treatment, whereas in the WT these enzymes need to be expressed and synthesized following UV treatment, potentially allowing more unrepaired DNA damage to accumulate.

On the other hand, for both mutant and WT strains, *recA* gene expression was only slightly upregulated by 5 min post treatment, but by 60 min was present at significantly higher levels than in untreated controls. The kinetics of *recA* upregulation in both strains suggests that *recA* is likely to be normally regulated independently of the *phrB*-*mutT* operon.

### Sensitivity of low light-adapted *Prochlorococcus* MIT9313 to UV light

**Fig. 5:**
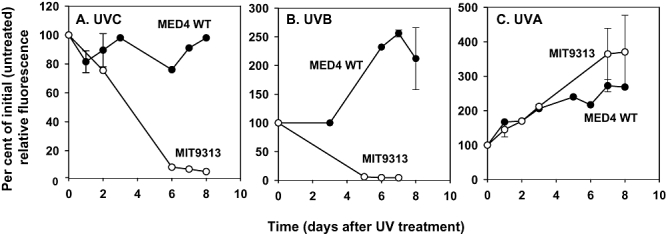
Sensitivity to UV of high light-adapted (MED4 WT) versus low light-adapted (MIT9313) strains of *Prochlorococcus*. Mid-log phase cells were treated with UV light for various time intervals (A: UVC for 30 s, B: UVB for 5 min, and C: UVA for 1 h) on day 0. Following UV treatment, cells were incubated under standard growth conditions and monitored for culture fluorescence over time. Fluorescence of UV-treated cultures is expressed as per cent of untreated culture fluorescence on day 0. Values are the mean of measurements of duplicate cultures, with error bars showing the standard deviation.•, MED4 WT; ○, MIT9313.

With the knowledge that low light-adapted strains of *Prochlorococcus* encode *mutT* but not *phrB,* and thus cannot synthesize photolyase (Kettler *et al.*, 2007), we tested the sensitivity of a low light-adapted strain (MIT9313) to UV exposure and compared it with MED4 WT (Fig. 5). As expected, MIT9313 was in fact considerably more sensitive to UVB and UVC radiation than was MED4 WT. This observation supports the contention that the photoreactivation system plays a significant role in UV resistance in the ocean, and suggests that the presence of *phrB* was selected for in the high light-adapted strain MED4 because it is a high light-adapted strain that occupies high light surface waters where UV radiation is present and can act as a selective agent. In contrast, photoreactivation repair would not be necessary in low light-adapted strains such as MIT9313, which occupy deeper, low light waters. The larger difference between the two strains seen with UVB radiation than with UVC radiation may reflect the fact that UVB radiation is generally less damaging than UVC, potentially allowing smaller dosage differences to be titrated more precisely.

The lack of differential sensitivity to UVA treatment at the doses used is consistent with the inability of UVA radiation to cause damage that can be repaired by photolyase (Sancar, 2004)

### Genome comparison of MED4 WT and MED4 UVR1

To determine the genomic underpinnings of the UVR phenotype we sequenced the genomes of both MED4 WT and MED4 UVR1. Genomes were first assembled using the sequence of a parent culture of MED4 (GenBank BX548174) as the reference, and then confirmed using a modified *de novo* assembly capable of detecting large insertions or deletions (*Experimental procedures*). MED4 WT contained 16 single nucleotide polymorphisms as compared with the published reference sequence (Rocap *et al.*, 2003, white rows in Table 2). The stability of the MED4 WT genome over more than 5 years of growth and serial transfers in the laboratory seems remarkable.

**Table 2:** DNA sequence differences between *Prochlorococcus* MED4 strains used in this study and the published parent MED4 genome (Rocap *et al.*, 2003).

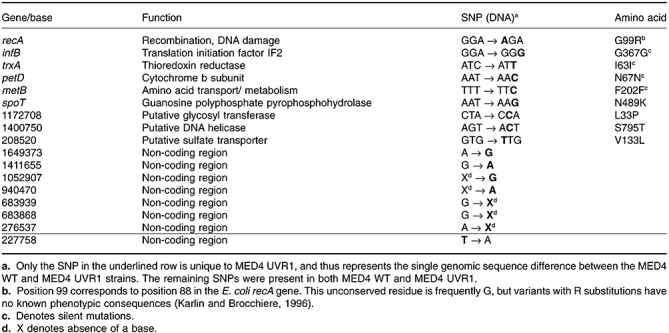

**Fig. 6:**
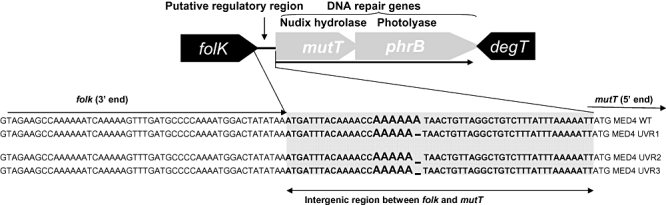
The MED4 *mutT*-*phrB* operon and location of UV hyper-resistance mutations in hyper-resistant mutants. The *mutT-phrB* operon of *Prochlorococcus* MED4 WT is flanked by two unrelated structural genes, *folK* and *degT* (http://www.microbesonline.org/cgi-bin/fetchLocus.cgi?locus=565621&disp=1). The highlighted sequence shows the intergenic region between *folk* and *mutT*, including the location of the homopolymer region (enlarged font) containing the 1 bp deletion (underlined) in the MED4 UVR strains as compared with MED4 WT. The intergenic region of MED4 WT was sequenced twice, first as the parent of MED4 UVR1, and then verified a year later as the parent of MED4 UVR2 and MED4 UVR3.

Relative to the reference sequence, MED4 UVR1 contained the same 16 SNPs as MED4 WT; in addition, however, it contained a single base pair deletion (underlined row, Table 2), located just upstream of the *mutT* gene, in a putative regulatory region of the *mutT*-*phrB* operon (Fig. 6). This result was confirmed by Sanger sequencing of a PCR fragment encompassing the 1 bp deletion (*Experimental procedures*), and strongly suggests that this mutation, located in a possible regulatory region for the downstream operon, is responsible for the high-level constitutive expression of the operon in the mutant, thus conferring UV hyper-resistance.

Because we do not have robust genetic tools for *Prochlorococcus*, we were unable to test directly whether this deletion would give rise to the UV-hyper-resist phenotype when transferred to a naïve WT strain. To further substantiate our hypothesis therefore we sequenced this region of the genome (containing the single base pair deletion in MED4 UVR1) in the other UV-hyper-resist strains, MED4 UVR2 and MED4 UVR3. Remarkably, both MED4 UVR2 and MED4 UVR3 carried the identical 1 bp deletion upstream of the *mutT*-*phrB* operon (Fig. 6). Although the mechanism by which this sequence may control expression of the operon is not clear, this result provides compelling evidence that the deletion is responsible for the UVR phenotype, as the strong UV challenge repeatedly enriched for cells containing the identical deletion mutation. Although there are no obvious regulatory motifs present in the intergenic sequence, and such sequences have not yet been defined for *Prochlorococcus*, a conceivable scenario is that a transcriptional activator or repressor might normally bind to the upstream region to regulate expression of the *mutT-phrB* operon in the WT case. The deletion in the mutant strain may disrupt the binding site, either preventing binding of the repressor, or causing the activator to bind more efficiently, resulting in constitutive expression. The role of *radA* in this model is unclear, as the function of its encoded enzyme remains to be elucidated. Additional studies of global transcription following UV treatment should clarify these issues further.

We note that MED4 UVR1 has not reverted to UV sensitivity despite being maintained under non-selective conditions for several years, which might be explained both by the fact that spontaneous compensatory 1 bp insertion mutations are relatively rare (Schaaper and Dunn, 1991; Mira *et al.*, 2001), and that MED4 WT does not appear to grow faster than the mutant under laboratory conditions.

### Analysis of the *mutT* genomic region in sequences from wild *Prochlorococcus*

Because increased resistance to UV radiation could conceivably confer an advantage to *Prochlorococcus* that dominate surface waters, it was of interest to determine whether the mutant sequence could be found in the wild. We searched the GOS (Global Ocean Survey) metagenomic database (*Experimental procedures*) to see whether we could find any incidence of the single base deletion in fragments of *Prochlorococcus* genomes that could be identified as coming from directly upstream of *mutT.* The search revealed 31 sequences with high similarity to MED4, one of which contained the SNP found in our mutant strains (JCVI_READ_1103359078035). Seven additional sequences contained ‘G’ in place of ‘A’ at the same position as the deletion mutation [i.e. AAAAGA instead of AAAA_A (mutant) or AAAAAA (WT) – see Table 3]. The remaining 23 sequences contained the WT sequence AAAAAA at the same location (Table 3). While there are not enough data to make statistical arguments, and we have not taken sequencing error into consideration – especially for this homopolymeric stretch of DNA – we find these data provocative albeit inconclusive with regard to the relevance of our laboratory observations to wild populations of *Prochlorococcus.* We also emphasize that the number of sequences analysed is vanishingly small relative to the 10^4^–10^5^*Prochlorococcus* cells ml^−1^ populating many of the stations sampled in GOS. Thus even if the mutation were present at a high frequency, we would not necessarily expect to see it in a sample of this size.

**Table 3:** Sequences upstream of the *mutT-phrB* operon in wild *Prochlorococcus* strains.

Strain or GOSa read number	Relevant sequence upstream of *mutT-phrB*
MED4 WT	AAAAAA
MED4 UVR1, UVR2, UVR3	AAAA_A
JCVI_READ_1103359078035	AAAA_A
JCVI_READ_1093017692266	AAAAGA
JCVI_READ_1093018630368	AAAAGA
JCVI_READ_1103769464916	AAAAGA
JCVI_READ_1103769440186	AAAAGA
JCVI_READ_1103769744552	AAAAGA
JCVI_READ_1103242864663	AAAAGA
JCVI_READ_1103359190125	AAAAGA
JCVI_READ_ 630691	AAAAAA
JCVI_READ_ 843287	AAAAAA
JCVI_READ_1519837	AAAAAA
JCVI_READ_1568009	AAAAAA
JCVI_READ_1653138	AAAAAA
JCVI_READ_1664920	AAAAAA
JCVI_READ_1091141237359	AAAAAA
JCVI_READ_1092256175212	AAAAAA
JCVI_READ_1092963601548	AAAAAA
JCVI_READ_1093017692266	AAAAAA
JCVI_READ_1092344011058	AAAAAA
JCVI_READ_1092347061561	AAAAAA
JCVI_READ_1092344012813	AAAAAA
JCVI_READ_1103769374878	AAAAAA
JCVI_READ_1102140312344	AAAAAA
JCVI_READ_1105333561959	AAAAAA
JCVI_READ_1103668709694	AAAAAA
JCVI_READ_1103242175527	AAAAAA
JCVI_READ_1105333435622	AAAAAA
JCVI_READ_1104230100283	AAAAAA
JCVI_READ_1103242847341	AAAAAA
JCVI_READ_1108840035938	AAAAAA
JCVI_READ_1108830123070	AAAAAA

a The GOS database can be accessed at http://camera.calit2.net/index.php.

## Discussion

Using visible light, especially in the blue-violet range, as a source of energy, photolyases catalyse the repair of cyclobutane dipyrimidine dimers formed by UV damage to DNA (Sancar, 2004). Metagenomics studies have shown that photolyase and cryptochrome gene homologues have been found to be highly represented in surface seawater (DeLong *et al.*, 2006; Frias-Lopez *et al.*, 2008; Singh *et al.*, 2009), in one instance, representing ∼0.1% of the genes present in a sample from a high-UV ocean environment (Singh *et al.*, 2009). Such environments are also enriched for transcripts of these genes (Frias-Lopez *et al.*, 2008). Results of these studies are consistent with the high levels of UV radiation to which the ocean surface is exposed daily, and which, according to recent measurements, may penetrate to depths of tens of metres (Tedetti and Sempere, 2006). Moreover, photolyase is encoded on the genomes of only a subset of the sequenced *Prochlorococcus* strains, specifically those that are high light-adapted or display increased relative fitness in fluctuating light (Kettler *et al.*, 2007). Thus photolyase, specifically needed to repair radiation damage to DNA, appears to be maintained when there is a strong selection for its activity. This strong selection is reflected by the increased sensitivity to UVB and C of low light-adapted strain MIT9313, which does not encode *phrB*, as compared with MED4 WT. Further, this difference in sensitivity between MIT9313 and MED4 WT was not observable in response to UVA radiation, which does not cause the formation of pyrimidine dimers. Whereas UVC and UVB can cause direct damage to DNA, UVA (320–400 nm) causes only indirect damage through reactive oxygen intermediates (Kidambi *et al.*, 1996).

Nudix hydrolase, on the other hand, is encoded by the ‘core’ gene *mutT,* found in all the *Prochlorococcus* genomes sequenced to date (Kettler *et al.*, 2007). This is consistent with its function to repair damage to GTP caused by reactive oxygen species formed not only as a result of oxidizing radiation such as UV light, but also formed as a result of oxidative stress from activities such as photosynthesis (Latifi *et al.*, 2009). It is noteworthy that although UVC radiation does not normally penetrate the earth's atmosphere, the levels of UVA and UVB radiation measured at the surface of the ocean and possibly tens of metres below may reach or greatly exceed the doses used in these experiments (Buma *et al.*, 2001).

Interestingly, photolyase and nudix hydrolase repair activities are not error prone and are thereforenon-mutagenic, in contrast to SOS repair (a form of dark repair) that allows survival at the cost of mutagenesis. Despite the fact that MED4 and other strains of *Prochlorococcus* encode a number of SOS-type genes including *recA* and *lexA* (the canonical repressor of SOS genes), the potential advantage of using error-free repair is apparent, as it would seem that the small, streamlined genomes of the high light-adapted ecotypes of *Prochlorococcus* would be adversely affected by high mutation rates. In support of this idea, *Prochlorococcus* strains also lack low-fidelity DNA polymerases II and IV, which are known to replicate across lesions, thereby leading to mutations (Napolitano *et al.*, 2000; Michel, 2005). They do, however, encode one of the low-fidelity DNA polymerases, PolV (encoded by *umuCD*). It is noteworthy that selection for resistance to repeated UV light exposure in *E. coli* has produced mutants that are typically affected in several different ‘dark repair’ genes involved in DNA replication and repair (Alcantara-Diaz *et al.*, 2004), including *radA uvrA*, and *polA,* whereas enrichment for UV hyper-resistance in MED4 resulted in a lesion affecting photoreactivation repair. Although the specific *E. coli* UV-resistance mutations isolated may at least partly be an artefact of their selection, i.e. they were selected in the dark in order to avoid photoreactivation repair following mutagenesis, it is tempting to speculate that with a larger and less streamlined genome, *E. coli* might be better able to tolerate mutations caused by error-prone repair mechanisms and may therefore make more use of these mechanisms.

Photolyase has been shown to be the major UV-resistance factor for another cyanobacterium, *Synechocystis* PCC 6803 (Ng and Pakrasi, 2001). This was shown genetically, i.e. a knockout of the *phrB* gene conferred increased UV sensitivity to the organism, whereas a *recA* knockout mutant was still relatively resistant to UVC (Minda *et al.*, 2005). The authors suggested that RecA may be used primarily for other functions in this organism, such as recombination repair, which is error-free (Sargentini and Smith, 1985; Cox, 1999). Although *Synechocystis* PCC 6803 also encodes a *lexA* gene, it may also be used for functions other than the regulation of SOS repair. Our data show that the *Prochlorococcus recA* gene was upregulated in both MED4 WT and MED4 UVR1 following UV irradiation, but only after the *phrB* and *mutT* genes were upregulated. It is not known whether MED4 or any *Prochlorococcus* strain makes use of an SOS repair system, and if so, under what circumstances. However, regardless of whether the 1 bp deletion was a new mutation caused by UV treatment or a pre-existing mutant in the original MED4 batch culture, it is striking that three enrichments for UV hyper-resistance, carried out at different times from the same MED4 WT strain using 10 rounds of treatment with UVC radiation of sufficient intensity to kill most of the cells, selected for this same 1 bp deletion mutation – perhaps suggesting there may be limited ways of becoming UV hyper-resistant. Further, a comparison of the genome sequences of MED4 WT and MED4 UVR1 points to high genome stability in the surviving cells. Again, with frequent and long-lasting environmental UV irradiation exposure, it would seem advantageous for *Prochlorococcus* to make use primarily of error-free photoreactivation repair systems, especially with the abundance of visible light available to power the system.

The role of an SOS repair system in *Prochlorococcus* has yet to be established. Future experiments that measure expression of relevant candidate SOS and other DNA repair genes under conditions designed to simulate exposures in the natural environment will address this question. In this context it is interesting to note that true temperate phage that can be excised from the bacterial genome require SOS-type processes, initiated by DNA damage, for their excision (Rokney *et al.*, 2008). To date, despite the isolation of many *Prochlorococcus* cyanophage and despite strong evidence of the presence of phage genes in both low and high light-adapted *Prochlorococcus* genomes, temperate phage have not yet been isolated for this organism. An increased understanding of the role of SOS and photoreactivation in *Prochlorococcus* DNA damage repair in low and high light-adapted strains may help to elucidate possibilities for lysogeny in *Prochlorococcus*.

## Experimental procedures

### Growth conditions

All *Prochlorococcus* cultures were grown at 22°C under constant light provided by cool-white fluorescent lamps at irradiances of ∼25 mol Q m^−2^ s^−1^ for MED4 strains, and ∼16 mol Q m^−2^ s for MIT9313. Cultures were grown in Pro99 medium, consisting of sterile (0.2 µm filtered, autoclaved) Sargasso Sea water supplemented with 800 µM NH_4_Cl, 50 µM NaH_2_PO_4_, and trace metals (Moore *et al.*, 2007). Growth was monitored using Turner Design fluorometer 10-AU to measure bulk chlorophyll fluorescence, used as a proxy for culture cell density (Moore *et al.*, 2007). Bulk chlorophyll fluorescence has been shown to be directly proportional to cell concentration for cells growing in exponential phase (Moore *et al.*, 2007), as determined by measuring cell number throughout log phase by means of flow cytometry.

### UV treatment and enrichment for hyper-resistant mutants

In all cases, UV treatment was applied using a UVLMS-38 8-W UV lamp (UVP, Upland, CA), generating wavelengths of 365/302/254 nm, with cells at a distance of 22 cm below the light source. Cells were slowly rotated using a mechanical rotator platform during all UV treatments. For resistance enrichment, cells were irradiated at room temperature for 30 s at 254 nm (UV fluence 9.2 J m^−2^) in an uncovered sterile glass Petri dish (150 mm diameter) containing 25 ml of culture grown to a fluorescence of ∼100 (early- to mid-log phase). Following treatment, cells were immediately returned to standard growth conditions. In cases where fluorescence decreased following UV treatment, cells were maintained under standard growth conditions until they recovered (i.e. reached a fluorescence of 100). After recovery cells were diluted 1:10 into Pro99 medium and again grown to fluorescence of ∼100 for the next round of UV irradiation. To confirm that culture fluorescence was proportional to cell number following UV treatment, cell counts on selected samples were carried out by means of flow cytometry, using 2 µm fluorescent beads as a size reference (Moore *et al.*, 2007). Results are shown in Fig. S1.

Once isolated, UV-hyper-resist strains were purified at least 20 times by dilution to very low titre (10 cells ml^−1^), followed by regrowth. This process was necessary because it is difficult, and in this case not possible, to isolate and regrow pure single colonies of *Prochlorococcus.* In addition, at least 10 cells ml^−1^ were required for these cultures to grow in liquid. However, DNA sequencing of mutant versus WT batch cultures confirmed the appropriate genotypes, i.e. all three mutant cultures contained the same deletion mutation upstream of the *mutT-phrB* operon. Furthermore, in spite of continuous culturing, involving cycles of dilution and regrowth, for periods longer than 2 years, the mutants have retained their UV-hyper-resistant genotype and phenotype.

### RNA preparation

Cells (UV-treated or untreated) were collected by centrifugation (7 min, 12 000 *g*, 22°C), resuspended in 1 ml of RNA later (Ambion, Austin, TX, USA), quick frozen and stored at −80°C. RNA was isolated using the Mirvana miRNA isolation kit (Ambion) according to the manufacturer's instructions. DNA was removed using Turbo DNase treatment (Ambion) according to the manufacturer's instructions and DNA removal was confirmed by PCR using the primers shown in Table 1 for qRT-PCR. RNA was then ethanol precipitated and resuspended to a concentration of 100 ng ml^−1^.

### qRT-PCR reactions

RNA was isolated as described above, reverse-transcribed, and subjected to triplicate real-time PCR reactions as described previously (Frias-Lopez *et al.*, 2008), using the primer sets shown in Table 1. We calculated gene expression levels using the Comparative C_T_ (2^−ΔΔCT^) method (Livak and Schmittgem, 2001; Frias-Lopez *et al.*, 2008), where C_T_ is the threshold cycle at which fluorescence rises above background levels, and ΔΔC_T_ = ΔC_T_, sample − ΔC_T_, reference. Expression of gene *rnpB* was used as the endogenous reference.

### Microarray hybridization and analysis

For microarray hybridizataion, 100 ng of RNA was first amplified using the MessageAmp II-Bacteria Kit (Ambion) according to the manufacturer's instructions, as previously described (Frias-Lopez *et al.*, 2008).

cDNA was synthesized, labelled, and hybridized to customized MD4-9313 microarrays (Affymetrix, Santa Clara, CA), and scanned, all as previously described (Lindell *et al.*, 2005). Hybridizations were visualized using GeneSpring software (version 7.3.1; Silicon Genetics, Palo Alto, CA), initially normalized using the Robust Multichip Average algorithm (Bolstad *et al.*, 2003) implemented in GeneSpring, and were later normalized using the Loess correction performed by using the open-source Statistical Data Analysis software R version 2.5.0 (http://www.r-project.org/). We used the program QVALUE, which measures significance in terms of the false discovery rate (Storey and Tibshirani, 2003). A gene was considered as differentially expressed if the *q*-value was < 0.05.

### Genome sequencing

#### Library preparation

Genomic DNA libraries suitable for sequencing on the Illumina Genome Analyzer were prepared as described (manufacturer's document ‘11251892_Genomic_DNA_Sample_Prep.pdf’) except that genomic DNA (30 µg in 300 µl TE, in an ice bath solution) was fragmented using a model UCD-200 Bioruptor sonicator (Diagenode): low power, 15 s on/15 s off cycle, for a total of 10 min. Adaptor–DNA constructs were purified on a 2% agarose-TAE gel, run at 120 V for 60 min, with a low-molecular-weight DNA ladder (New England BioLabs) run in parallel. Blank wells between each sample prevented DNA cross contamination. DNA of size 150–200 bp was excised from the gel and purified using a QIAGEN Gel Extraction Kit. Samples were eluted into 50 µl elution buffer, then extracted using the Qiagen MinElute Gel Extraction Kit and eluted into 30 µl of elution buffer.

Purified adaptor–DNA constructs were enriched for fragments having the proper adaptor molecules on both ends using PCR (1 µl DNA, 25 µl Phusion DNA polymerase (Finnzymes Oy), 1 µl each of PCR primers 1.1 and 2.1 (© 2006 and 2008 Illumina), and 22 µl water). The PCR protocol was: 30 s at 98°C, 18 cycles of: 10 s at 98°C, 30 s at 65°C, 30 s at 72°C, followed by a final extension of 5 min at 72°C. Amplified DNA was purified using the Qiagen QIAquick PCR Purification Kit, and eluted into 50 µl of elution buffer. The DNA library concentration was determined using a NanoDrop ND8000 spectrophotometer, followed by analysis on an Agilent 2100 Bioanalyzer using the Agilent RNA 6000 Pico Chip.

#### Sequencing methods

DNA library samples were diluted to a concentration of 10 nM, and were clustered at a final concentration of 2.0 pM each on an Illumina GA flow cell, according to the manufacturer's standard protocols. Sequencing of the samples on the clustered flow cell was performed on the Genome Analyzer using a single read 36 cycle protocol, following the manufacturer's standard procedures and protocols.

#### Data analysis

Each sample (MED4 WT and MED4 UVR1) was run on one flow cell lane of an Illumina GA I, yielding 5.8 and 7.8 million 36-base sequence reads respectively. Raw images were processed using the Genome Analyzer Pipeline Software v0.3 to produce DNA sequence files in FASTQ format. Output files were imported and assembled using the SeqMan NGEN v1.2 program from DNAStar with the original published MED4 genome (BX548174) as the reference sequence. To check for possible large insertions, deletions, inversions, or genomic rearrangements, further assembly analysis was carried out using NGen's Split Reference Sequence Strategy (http://www.dnastar.com/products/ReferenceGuidedAssembly.php). Resulting genome assemblies were imported into DNAStar LaserGene8, where mutations were identified.

### Sequencing of PCR fragments

DNA fragments spanning the putative regulatory region upstream of *mutT* were PCR-amplified using forward and reverse primers: (5′-GGTGGGAAGATTTTTATACGGTTGACGAG-3′, and 5′-AATGCTGCGCCAGGATGC-3′) respectively. Amplified DNA fragments were treated with ExoSAP-IT (USB, Cleveland, OH) and submitted for pyrosequencing (Amplicon Express, Pullman, WA).

### Analysis of GOS database

The GOS database was searched to recruit reads and their paired ends homologous to a 30 bp stretch of the 51 bp intergenic region (Fig. 6) between *folK* and *mutT*. This search yielded 175 sequences that matched at least 30 bp of the intergenic region. To select those with high similarity (at least 95%) to high-light *Prochlorococcus* strains over the entire length of the read (normally ∼700–1000 bp), blastn was used to compare the reads against the microbial genomes database in Microbes Online (http://www.microbesonline.org). Thirty-one reads had high similarity to *Prochlorococcus*, and these were analysed for the presence of SNPs in the intergenic region, as described above. Reads of interest can be accessed in the CAMERA (Community Cyberinfrastructure for Advanced Marine Microbial Ecology Research and Analysis) database (http://camera.calit2.net/index.php).

## Supporting information

**Fig. S1.** Cell fluorescence following UV treatment is proportional to cell number. Cells from mid-log phase cultures of MED4 WT and MED4 UVR1 were subjected to 30 s of UV exposure (254 nm) on Day 0, then returned to standard growth conditions. Untreated (○) and UV-treated (•) cultures were sampled for culture fluorescence (A and B), and for cell number using flow cytometry (C and D). Values are the mean of measurements of duplicate cultures, with error bars showing the standard deviation.
