# Supplementary figures and images for: UV hyper-resistance in Prochlorococcus MED4 results from a single base pair deletion just upstream of an operon encoding nudix hydrolase and photolyase
Source: Environ Microbiol. 2010 Jul;12(7):1978–88. doi: 10.1111/j.1462-2920.2010.02203.x (PMC2955971; doi:10.1111/j.1462-2920.2010.02203.x)

## Slide 1
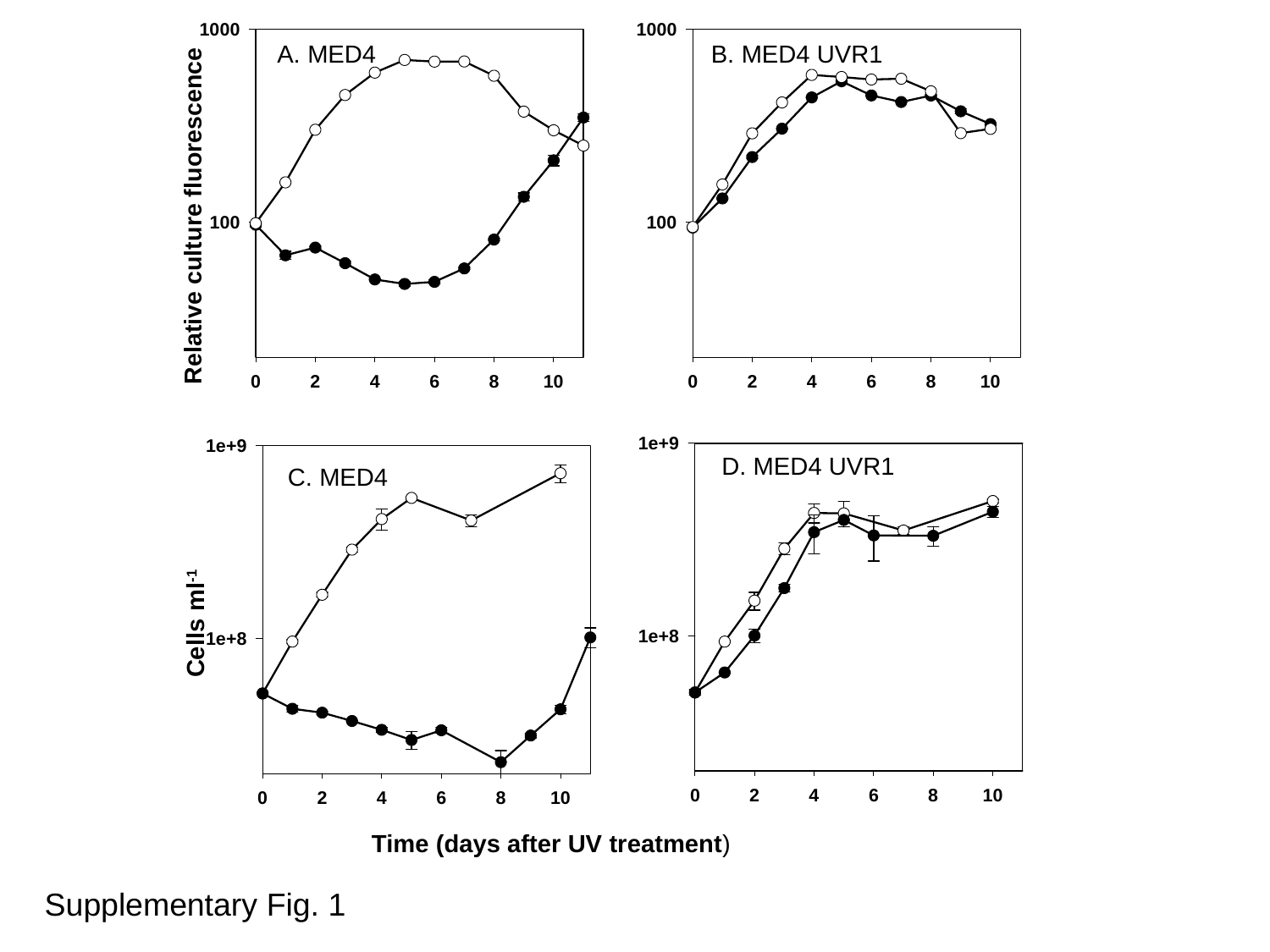

A. MED4
B. MED4 UVR1
D. MED4 UVR1
C. MED4
Cells ml-1
Time (days after UV treatment)
Supplementary Fig. 1

Supplement: Supplementary file 1 [file emi0012-1978-SD1.ppt]
